# Prognostic Significance of Platelet-to-Lymphocyte and Neutrophil-to-Lymphocyte Ratios for Complications of Diabetic Foot Ulcers: A Meta-Analysis

**DOI:** 10.7759/cureus.112920

**Published:** 2026-07-18

**Authors:** Dafaalla Salih, Elfa Obaied Elhag Elabas, Razan Imad Mohamedtahir Fadlalla, Aseel Osama Abdelrahman Dafaalla, Hani Bilal, Alaa Kamal Hassan Hamedelnile, Omer A Abdelrahman, Eltayeb Elmamoun, Mona Waheeb Khalifa Rahamt Alla, Afnan Ali

**Affiliations:** 1 College of Medicine, Alzaiem Alazhari University, Khartoum, SDN; 2 General Surgery, Nile University, Khartoum, SDN; 3 Pediatric Surgery, University of Khartoum, Khartoum, SDN; 4 Surgery, University of Khartoum, Khartoum, SDN; 5 Internal Medicine, University of Khartoum, Khartoum, SDN; 6 Surgery, Nahda College, Khartoum, SDN; 7 Surgery, Ahfad University for Women, Omdurman, SDN; 8 General and Laparoscopic Surgery, Omdurman Islamic University, Omdurman, SDN

**Keywords:** diabetic foot ulcer, inflammation, leg amputation, nlr, plr

## Abstract

One of the main consequences of diabetes mellitus is diabetic foot ulcers (DFUs), which often result in amputation and death. Two inflammatory biomarkers, platelet-to-lymphocyte ratio (PLR) and neutrophil-to-lymphocyte ratio (NLR), measured in standard blood testing, have become potential indicators of many adverse outcomes in diabetes-related complications. However, evidence regarding their association with complications of DFUs remains limited and inconsistent.

This study critically reviewed the prognostic value of both markers in predicting clinical consequences amongst patients with DFU. An inclusive literature search across PubMed, Web of Science, Embase, and ProQuest identified 22 relevant studies. Meta-analysis showed that NLR was associated with amputation (standardized mean difference (SMD) 0.78, p = 0.002) and osteomyelitis (SMD 0.31, p = 0.001). Similarly, PLR was associated with amputation (SMD 0.29, p = 0.005) and osteomyelitis (SMD 0.40, p < 0.001). Pooled diagnostic performance for mortality prediction showed good accuracy for NLR (area under the curve (AUC) 0.83) and moderate accuracy for PLR (AUC 0.75). Overall, both NLR and PLR are promising and readily available biomarkers for risk stratification in DFU patients. The results emphasize the necessity of standardized cut-off values and prospective validation.

## Introduction and background

Diabetic foot ulcers (DFUs) are among the most serious complications of diabetes mellitus, affecting approximately 6.3% of patients globally, with a lifetime risk estimated at 19%-34% [1]. DFUs contribute substantially to morbidity, healthcare costs, and reduced quality of life due to their multifactorial pathogenesis, which includes peripheral neuropathy, peripheral arterial disease, infection, and impaired wound healing [1]. Among patients who develop DFUs, approximately 20% are at risk of lower-extremity amputation, and nearly 10% may die within one year of diagnosis [1,2]. Furthermore, the five-year mortality rate following the onset of DFUs ranges from 25% to 60%, exceeding that of several common malignancies [3].

The complications associated with DFUs are extensive and include functional decline, poor quality of life, severe infections, prolonged or recurrent hospitalizations, lower-extremity amputation, delayed wound healing, high recurrence rates, and increased mortality [4,5]. In addition, revision surgeries are frequently required following diabetic amputations, with many patients undergoing multiple procedures over time.

Chronic inflammation plays a central role in the progression of DFUs [6]. The underlying pathophysiology is complex and involves persistent inflammation, peripheral neuropathy, peripheral vascular disease, and impaired tissue regeneration. Emerging evidence also highlights the role of immunological dysregulation, including neutrophil dysfunction and impaired regulatory T-cell activity, in sustaining chronic, non-healing wounds [6].

Recently, systemic inflammatory indices derived from routine complete blood counts have gained attention as potential prognostic biomarkers. Among these, the neutrophil-to-lymphocyte ratio (NLR) - calculated as the ratio of circulating neutrophils to lymphocytes - and the platelet-to-lymphocyte ratio (PLR) - defined as the ratio of platelet count to lymphocyte count - reflect the balance between pro-inflammatory processes and immune regulation. Elevated NLR indicates a shift toward neutrophil-mediated innate inflammation and reduced lymphocyte-mediated adaptive immunity, whereas increased PLR reflects heightened platelet activation alongside relative lymphopenia. Both indices are associated with systemic inflammatory states [7].

These biomarkers are inexpensive, widely available, and easily reproducible, as they are derived from standard laboratory tests. They have been increasingly investigated as prognostic indicators across various clinical conditions. In patients with diabetes, elevated NLR and PLR have been associated with an increased risk and severity of complications, including microvascular conditions such as nephropathy and retinopathy, as well as macrovascular diseases such as lower-extremity arterial disease. Higher levels of these indices have also been linked to systemic inflammation and increased mortality risk [8-10].

Although several studies have examined the association between NLR, PLR, and DFU-related outcomes, the findings remain fragmented and inconsistent. Therefore, a comprehensive synthesis of the available evidence is warranted. This review aims to evaluate the clinical utility of NLR and PLR as prognostic markers for adverse outcomes in patients with DFUs.

## Review

Methods

Search and Selection Process

The review followed the specifications of the PRISMA statement [11]. The PROSPERO registry included the review protocol (CRD420251238315). A comprehensive search of PubMed, Embase, Web of Science, and ProQuest was conducted on 15th March 2026. The search strategy combined free-text keywords related to the exposures and outcomes. For example, the PubMed search strategy included the following terms, which were applied to titles and abstracts: (“platelet to lymphocyte ratio” OR “platelet-lymphocyte ratio” OR PLR OR “neutrophil to lymphocyte ratio” OR “neutrophil-lymphocyte ratio” OR NLR) AND (“diabetic foot ulcer” OR DFU OR “diabetic foot” OR “diabetic septic foot” OR “foot ulcer”). No restrictions were applied regarding publication date or population characteristics. Moreover, we screened the references of the included studies for relevant studies. All records were imported into the Rayyan website for screening [12].

Study selection was conducted in two stages. First, titles and abstracts were screened to identify potentially relevant studies. This was followed by a full-text review to assess eligibility based on the predefined inclusion and exclusion criteria.

Studies were considered eligible if they met the following criteria: the study population included adult diabetic patients presenting with DFU; the exposures of interest were NLR and PLR; the reported outcomes involved DFU-related complications; and the study design was observational in nature, including cohort, case-control, or cross-sectional studies. Exclusion criteria included case reports, reviews, editorials, conference abstracts, non-English publications, and studies that did not report sufficient data on the outcomes or exposures of interest.

Data Extraction and Quality Assessment

A standardized extraction form was used by two independent reviewers, with conflicts resolved by a third reviewer. Extracted data from studies included study characteristics (author, year, country), sample size, patient demographics, biomarker type (NLR, PLR, or both), and reported outcomes of DFU complications. Effect estimates' means and standard deviations (SDs), along with area under the curve (AUC) values, were recorded. The level of quality of each of the examined studies had been assessed according to the QUIPS standard tool, which judged research participation and turnover, prognostic and outcome variables, study bias, analysis of data, and presentation [13]. The certainty of evidence for each pooled outcome was assessed using the Grading of Recommendations Assessment, Development and Evaluation (GRADE) approach, which was adapted for prognostic factor research. Certainty was rated across the domains of risk of bias, inconsistency, indirectness, and imprecision and was upgraded when a large magnitude of association was observed; the publication bias domain was not scored given the inability to formally test for it.

Statistical Analysis

Meta-analysis was conducted to quantitatively examine the association between NLR, PLR, and outcomes. Continuous outcomes were pooled using standardized mean differences (SMDs). Accuracy for mortality prediction was evaluated via the pooled area under the receiver operating characteristic curve. A random-effects model was applied in the analyses. Statistical heterogeneity was further explored qualitatively based on a detailed leave-one-out sensitivity analysis after the initial assessment with the I² statistic. Due to the limited number of studies in some analyses, a formal assessment of publication bias was not possible. The analyses were completed in the meta-package of R version 4.3.2 (The R Foundation for Statistical Computing, Vienna, Austria).

Results

*Study Selection* 

**Figure 1:**
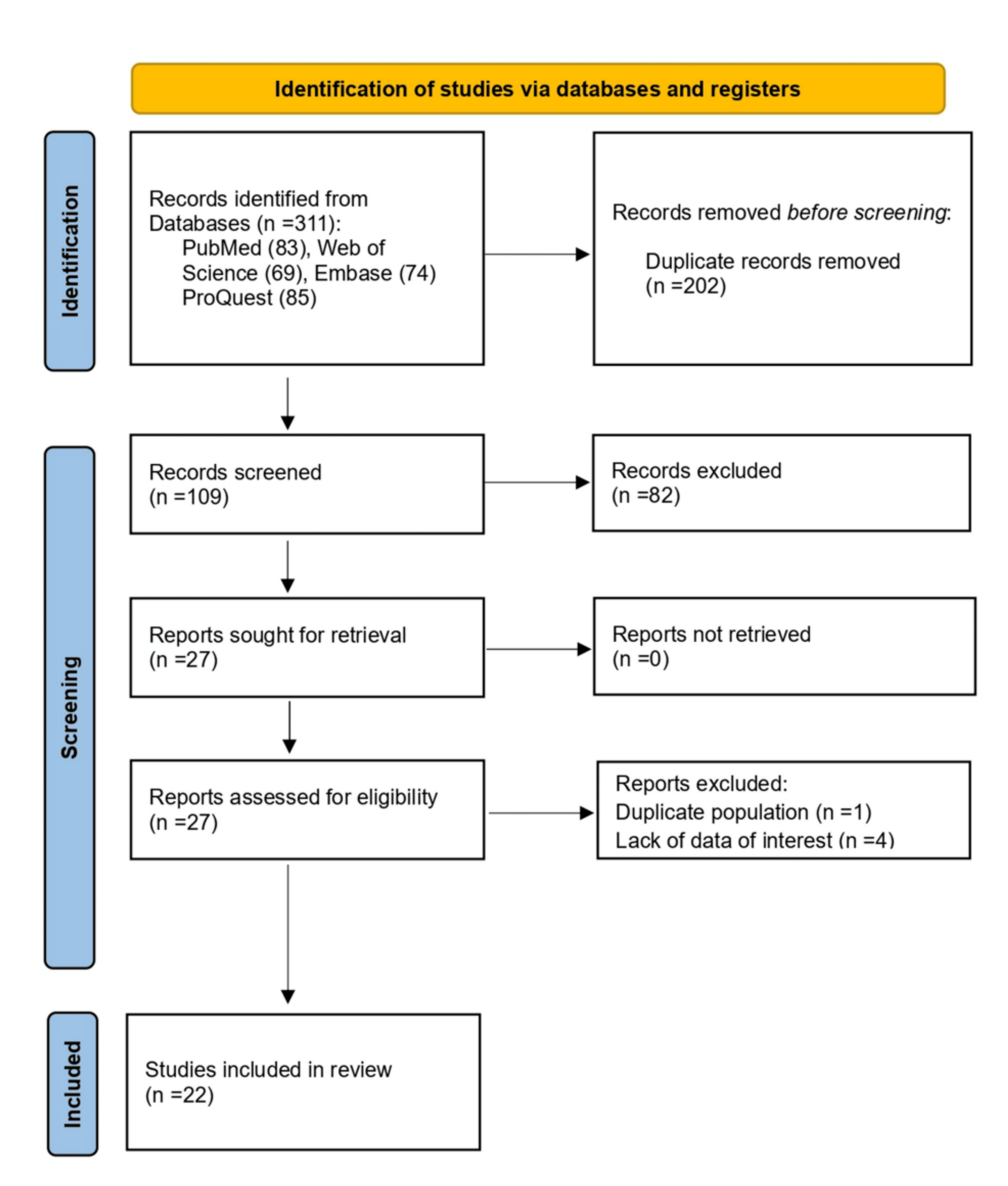
PRISMA flow diagram illustrating the study selection process for the systematic review and meta-analysis The figure summarizes the identification, screening, eligibility assessment, and inclusion of studies, including the number of records identified from each database, duplicates removed, records excluded during title/abstract and full-text screening, and the final studies included in the qualitative and quantitative syntheses.

The database search identified a total of 311 records. After removal of duplicates, the remaining studies underwent title and abstract screening. Of these, 27 articles were selected for full-text review. During this stage, five studies were excluded due to insufficient data or overlapping study populations. Ultimately, 22 studies met the inclusion criteria and were included in the final analysis (Figure 1) [14-35].

**Figure 2:**
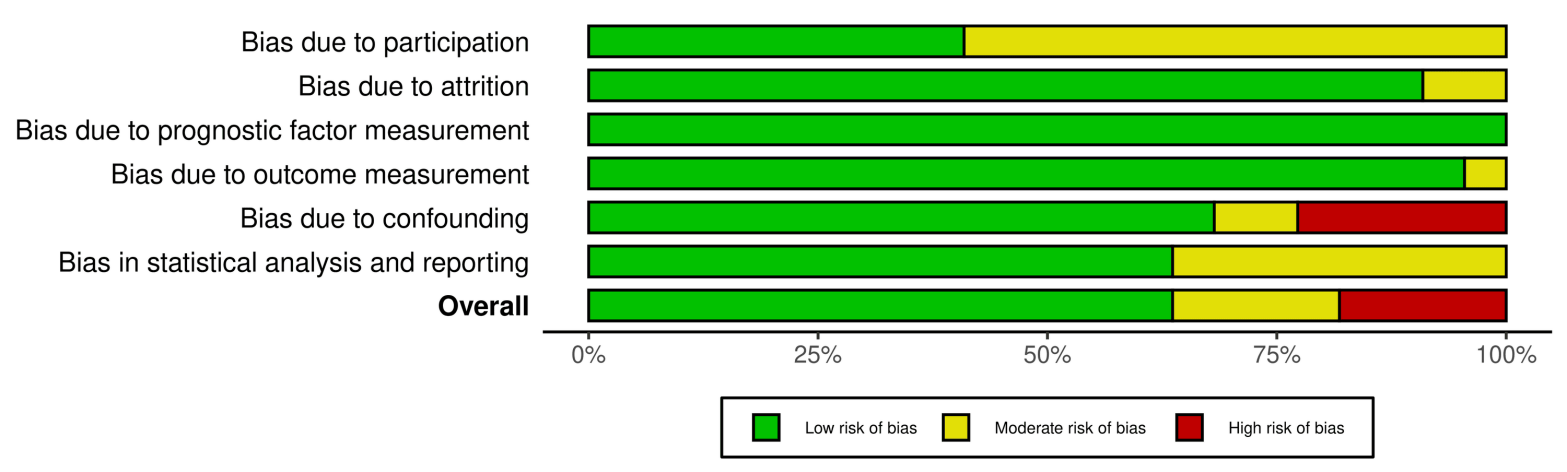
Risk of bias assessment Risk of bias assessment was performed based on the QUIPS instrument, which was used to examine the quality of the included studies by appraising study participation and attrition, prognostic factors and outcome measurements, study confounding, and statistical analysis and reporting.

The included studies represented diverse geographic regions, including Asia, Europe, and the Middle East, with sample sizes ranging from 47 to 688 participants. All studies evaluated the prognostic value of NLR, while 13 studies [14,16,18-20,22-25,28,29,33,34] examined PLR or both biomarkers. The outcomes assessed across studies included lower-limb amputation, mortality, osteomyelitis, and measures of DFU severity and healing (Table 1). Risk of bias assessment indicated that the methodological quality of the included studies was generally favorable (Figure 2). Most studies were judged to be at low risk of bias for prognostic factor measurement, study attrition, and outcome measurement. In contrast, study participation and statistical analysis/reporting exhibited a greater proportion of studies with moderate risk of bias. Bias due to confounding was the domain with the greatest methodological concern. Overall, the majority of the included studies were judged to have a low risk of bias. Applying the GRADE approach, certainty of evidence was rated as low for the associations between NLR and amputation, NLR and osteomyelitis, PLR and amputation, and PLR and osteomyelitis, and as moderate for the associations between PLR and mortality and PLR and mortality (Appendix 1). Across all quantitatively pooled outcomes, certainty was downgraded for risk of bias, driven predominantly by the participation and confounding domains described above. The PLR-amputation and NLR-amputation associations were upgraded for a large magnitude of association, which balances one level of downgrading in each case.

**Table 1:** Summary of baseline characteristics of the included studies populations AUC: area under the curve; NLR: neutrophil-to-lymphocyte ratio; PLR: platelet-to-lymphocyte ratio; PAD: peripheral arterial disease; DFU: diabetic foot ulcer

Study ID	Year	Country	Sample size	Age	Biomarker	Outcomes assessed	Results summary
Afshar et al. [14]	2025	Iran	126	60.81 ± 11.21	NLR and PLR	Amputation	Increased likelihood of requiring amputation in patients with DFUs when NLR > 6.08 (OR of 13.09, 95% CI: 5.143-33.32) and PLR > 210 (OR: 2.231, 95% CI: 1.066-4.669)
Arıcan et al. [15]	2020	Turkey	250	60 (55-65)	NLR	Amputation	NLR cut‑off value for prediction of major amputation was NLR >6.73 (AUC = 0.864, with 77% sensitivity and 76.5% specificity)
Ozer Balin et al. [16]	2022	Turkey	247	62 (28-95)	NLR and PLR	Diabetic Foot Osteomyelitis	NLR and PLR were significantly higher in osteomyelitis patients
Chen et al. [17]	2025	China	688	63 (54, 71)	NLR	Mortality	Higher NLR (> 4.22) is substantially linked with sudden death (HR: 3.64, 1.21-10.91)
Chen et al. [18]	2021	China	348	65.37 ± 9.61	NLR	Mortality	High NLR (> 2.76) and high PLR (> 160.05) are independent predictors of mortality. NLR showed higher sensitivity (AUC 0.679) than PLR (AUC 0.598)
Cosarca et al. [19]	2021	Romania	203	66.5 (62‑70)	NLR and PLR	Amputation	The NLR (above 3.485) was for predicting PAD (sensitivity 60.42%; specificity 72.44%)
Coşkun et al. [20]	2024	Turkey	386	59.62 ± 13.97	NLR and PLR	Amputation	No significant association between NLR, PLR, and amputation
Cunanan et al. [21]	2025	Philippines	280	57.01 ± 10.72	NLR	Amputation	With an optimum threshold of 9.33, analysis using ROC yielded an AUC = 0.8234 with high sensitivity and specificity. Also, NLR independently predicted major amputation (adjusted OR 1.48, 1.14-1.92).
Demirdal et al. [22]	2018	Turkey	280	59.5 ± 11.1	NLR and PLR	Osteomyelitis, PAD, and amputation	NLR >6.5 predicts PAD; PLR >187.3 predicts osteomyelitis. Both predictive of amputation.
Eren et al. [23]	2020	Turkey	78	57-59.9	NLR	Hospital Stay and Cost	PLR is an independent predictor of hospital cost. NLR/PLR increases with Wagner grade
Farine et al. [24]	2024	Italy	200	67.64 ± 11.23	NLR and PLR	Amputation and mortality	Significant positive correlation between high NLR/PLR and both amputation and mortality
Günay and Ekici [25]	2021	Turkey	87	70.63 ± 1.27	NLR and PLR	Mortality	NLR and PLR were associated with early mortality after amputation. For NLR, the AUC is 0.746 (threshold: 6.37). For PLR, the AUC is 0.547 (threshold: 247.28) (61% sensitivity, 54% specificity)
Jayanti et al. [26]	2025	Indonesia	50	54.36 ± 10.86	NLR	Amputation	ROC analysis of the NLR > 7.82 cut-off value for predicting amputation showed that the AUC = 0.908 (0.822-0.989). Patients with NLR > 7.82 had a higher risk of amputation
Metineren et al. [27]	2017	Turkey	56	72.73 ± 10.53	NLR	Mortality	No significant association between NLR and mortality
Nagarjan et al. [28]	2026	India	285	55.95 ± 10.42	NLR and PLR	Mortality	NLR > 9.1 (AUC 0.80) and PLR > 302.8 (AUC 0.686) are independent predictors of in-hospital mortality
Sales et al. [29]	2025	Philippines	535	57	NLR and PLR	Mortality	Cut-offs of 7.27 for NLR and 32.40 for PLR accurately predict in-hospital morbidity/mortality with 98% sensitivity
Sezen et al. [30]	2025	Turkey	77	62.6 ± 11.9	NLR	Amputation	No significant association between NLR, PLR, and amputation
Vincent et al. [31]	2024	Indonesia	179	54 years (median)	NLR	Amputation	NLR is an independent predictor of amputation (OR 1.04)
Xu et al. [32]	2023	China	156	18+ years	NLR	Healing, Amputation, Mortality	NLR (AUC 0.901) is a reliable indicator; higher values correlate with amputation and death
Yang et al. [33]	2025	China	202	61.98 ± 9.48	NLR and PLR	DFU Severity and Healing	Higher PLR correlated with a higher Wagner's grade and longer healing time
Zhang et al. [34]	2021	China	296	52.1 ± 9.3	PLR	DFU Severity	PLR is an independent risk factor (OR 1.029). Optimal critical value for predicting DFU is 147.6
Zhu et al. [35]	2023	China; Shanghai Univ. of TCM	389	68 years (median)	NLR	Amputation-free survival	High NLR is an independent risk factor (OR 3.811) for amputation.

NLR and Clinical Outcomes

**Figure 3:**
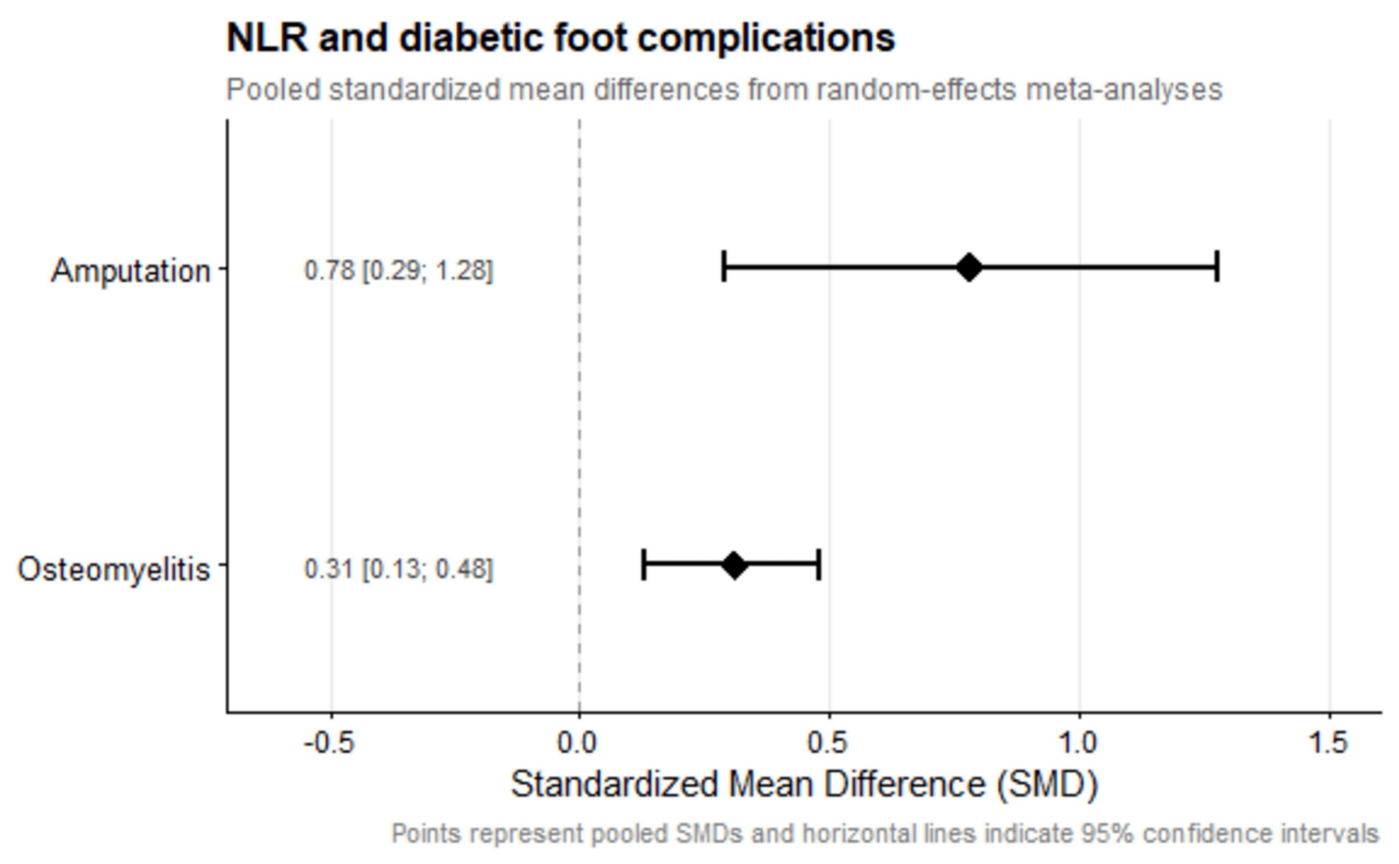
Summary meta-analysis of pooled SMDs in NLR between patients with and without diabetic foot complications Separate random-effects meta-analyses were performed for each complication. Squares represent the effect estimate for each study, with the size of the square proportional to the study weight, and horizontal lines indicate the 95% CIs. Diamonds represent the pooled SMDs with corresponding 95% CIs. Positive SMD values indicate higher NLR levels among patients with diabetic foot complications. SMD: standardized mean difference; NLR: neutrophil-to-lymphocyte ratio

Pooled analysis demonstrated a consistent relationship between elevated NLR and infectious complications in DFU patients (Figure 3). The NLR was associated with a high risk of amputation, with an SMD of 0.78 (95% CI 0.29-1.28, p = 0.002, I² = 92.6%). Similarly, NLR was significantly associated with osteomyelitis, as the results showed an SMD of 0.31 (95% CI 0.13-0.48, p = 0.001, I² = 0.0%).

PLR and Clinical Outcomes

**Figure 4:**
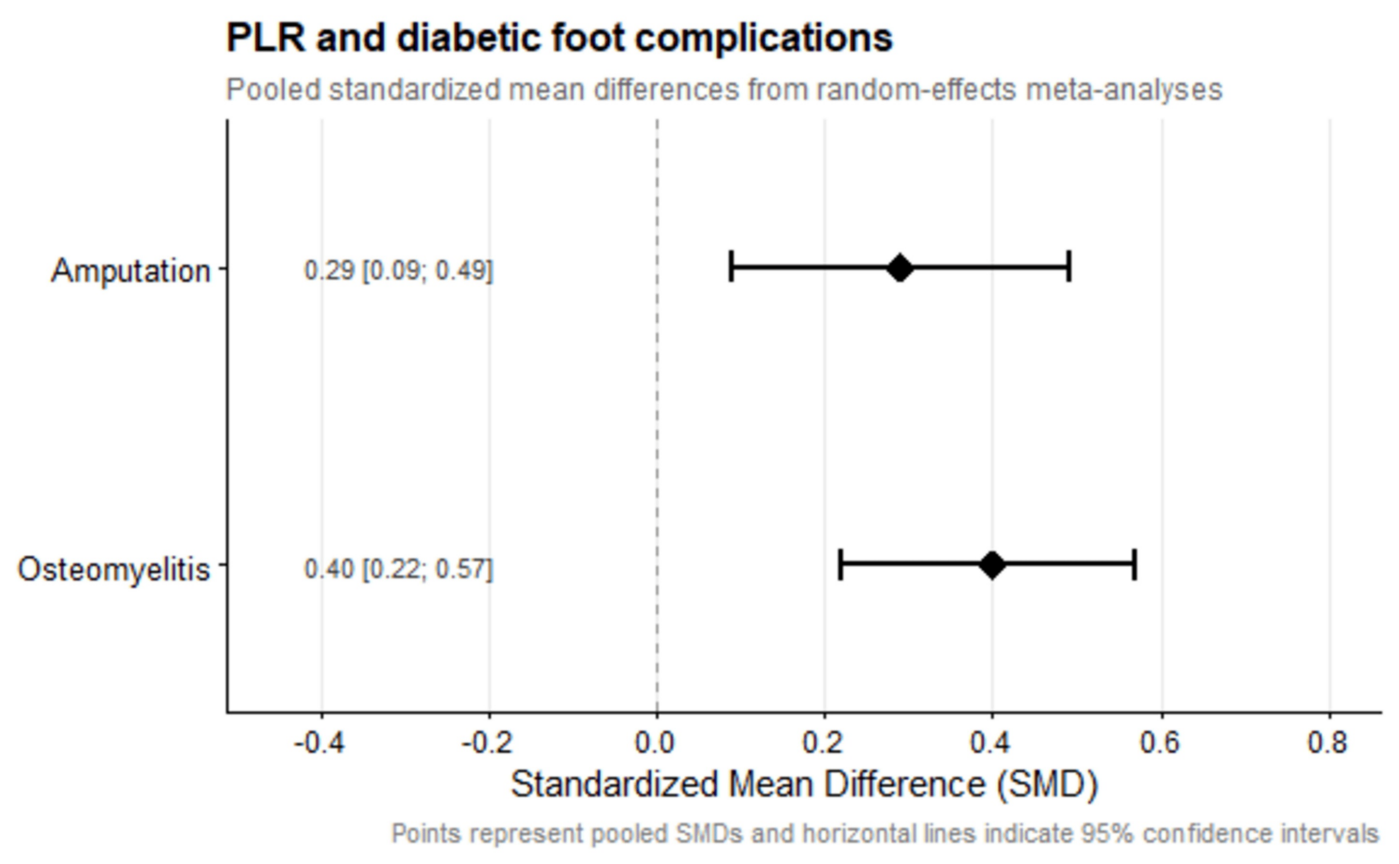
Summary meta-analysis of pooled SMDs in PLR between patients with and without diabetic foot complications Separate random-effects meta-analyses were performed for each complication. Squares represent the effect estimate for each study, with the size of the square proportional to the study weight, and horizontal lines indicate the 95% CIs. Diamonds represent the pooled SMDs with corresponding 95% CIs. Positive SMD values indicate higher PLR levels among patients with diabetic foot complications. SMD: standardized mean difference; PLR: platelet-to-lymphocyte ratio

With an SMD of 0.29 (95% CI 0.09-0.49, p = 0.005, I² = 44.4%), the pooled data for PLR showed a significant correlation with amputation. In addition, PLR was significantly associated with osteomyelitis, with an SMD of 0.40 (95% CI 0.22-0.57, p < 0.001, I² = 0.0%) (Figure 4).

Mortality Prediction

**Figure 5:**
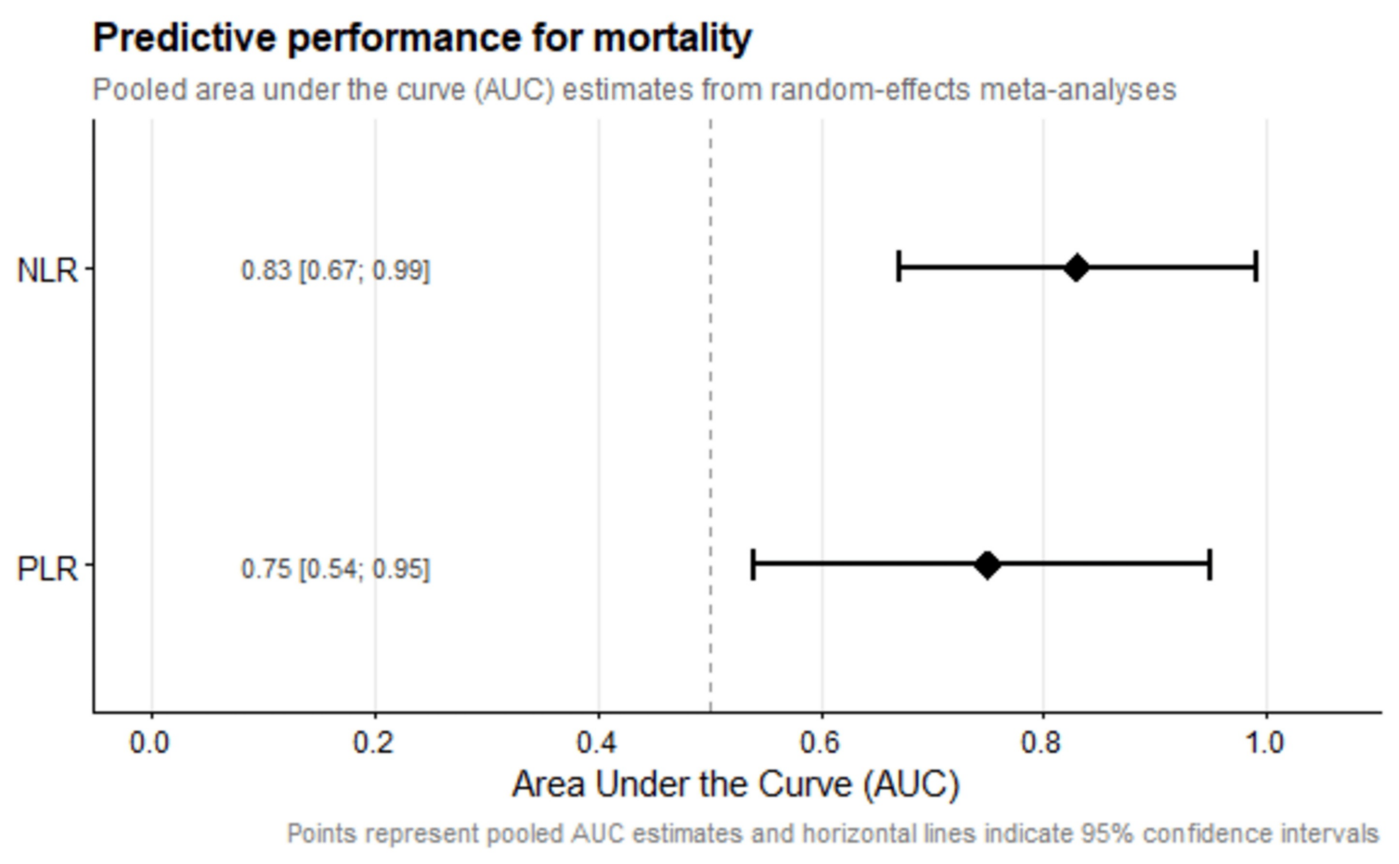
Summary meta-analysis showing the pooled AUC estimates for NLR and PLR in predicting mortality among patients with diabetic foot ulcers Individual study AUCs are presented with their 95% CIs. Higher AUC values indicate better discriminative ability for mortality prediction. AUC: area under the curve; NLR: neutrophil-to-lymphocyte ratio; PLR: platelet-to-lymphocyte ratio

The diagnostic performance of NLR and PLR for predicting mortality was evaluated using pooled AUC estimates. NLR demonstrated satisfactory predictive accuracy, as the result of the analyzed AUC was 0.83 (95% CI 0.67-0.99). PLR, on the other hand, had a value of 0.75 (95% CI 0.54-0.95), corresponding to moderate predictive power (Figure 5).

Leave-one-out sensitivity analyses were conducted for all meta-analyses to assess the influence of individual studies on the pooled estimates. Sequential exclusion of each study did not alter the magnitude, direction, or statistical significance of the pooled effect estimates for any outcome, including NLR and PLR associations with amputation, osteomyelitis, and mortality, as well as the pooled AUC analyses for mortality (Appendix 1).

Discussion

This review demonstrates that both examined markers are associated with adverse outcomes in patients with DFU. NLR, in particular, showed stronger and more consistent associations across outcomes, including amputation, osteomyelitis, and mortality.

Based on the results, elevated NLR appears to show a more consistent association with adverse outcomes in patients with DFUs than PLR across the included studies. However, this finding should be interpreted with caution given the substantial between-study heterogeneity observed in several analyses. Across multiple studies, elevated NLR is strongly associated with an increased risk of amputation, with optimal cut-off values ranging from 3.49 to 9.33. For instance, Afshar et al. [14] found that an NLR >6.08 conferred an odds ratio of 13.09 for amputation, while Cunanan et al. and Jayanti et al. reported high diagnostic accuracy (AUCs of 0.823 and 0.908, respectively) [21,26].

PLR shows more variable utility. While some studies report that elevated PLR predicts amputation and osteomyelitis, others demonstrate no significant association with amputation [14,35]. PLR has demonstrated predictive value for mortality in certain studies, though often with lower sensitivity and AUC values compared to NLR. Additionally, PLR correlates with DFU severity, healing time, and hospital costs [23,32,33]. Overall, both markers emerge as frequently reproducible biomarkers across diverse populations and outcomes.

The contribution of systemic inflammation to DFU progression supports the biological plausibility of these findings [8]. High neutrophil counts indicate acute inflammation, while lymphopenia indicates impaired immune regulation. Similarly, platelet activation contributes to thrombosis and microvascular dysfunction, further worsening tissue ischemia [14]. Lymphocytes are important mediators of humoral and cellular immune responses and are involved in immune memory and immune surveillance. The NLR is a simple and rapid method for assessing immune function and systemic inflammation [8]. Therefore, increased NLR and PLR indicate chronic low-grade inflammation, which is known to contribute to the pathogenesis and progression of DFU.

Both NLR and PLR were most strongly and consistently associated with amputation, one of the most severe morbidity-related endpoints. Amputation represents the end stage of established macrovascular compromise and uncontrolled infection, and the marked NLR elevation observed in these patients likely reflects an acute, neutrophil-driven inflammatory surge coupled with lymphocyte-mediated immunosuppression once infection has progressed to a limb-threatening stage. Concurrently, elevated PLR is thought to reflect platelet-driven microvascular thrombosis, compounding tissue ischemia [14,21,26].

In contrast, the association with osteomyelitis, although numerically smaller, was the most homogeneous of all outcomes examined. Osteomyelitis is a localized, infection-specific process in which neutrophil recruitment targets bacterial invasion of bone; the resulting NLR elevation therefore likely reflects a more direct marker of active regional infection rather than the broader systemic inflammatory burden implicated in amputation or mortality [22]. Evidence supports the diagnostic utility of NLR in osteomyelitis, with significantly higher levels observed in affected patients and independent predictive value demonstrated in multivariable analyses. Moreover, NLR correlates with the overall inflammatory burden and may be further elevated in more severe or pathogen-specific infections, supporting its role as a marker of active infection and disease severity [36].

Because mortality integrates the combined burden of infection, ischemia, and comorbidity, elevated NLR and PLR in this context are best interpreted as global severity markers, similar to their established prognostic role in critical illness.

From a clinical perspective, NLR and PLR are simple, inexpensive, and widely available biomarkers that may complement routine clinical assessment for risk stratification, particularly in identifying patients at increased risk of adverse outcomes such as amputation. However, the absence of standardized and externally validated cut-off values currently limits their clinical implementation. The thresholds used to define elevated NLR and PLR varied considerably across the included studies, reflecting differences in patient populations, disease severity, study design, and analytical approaches. This variability may have contributed to the between-study heterogeneity observed in several meta-analyses and could also influence the magnitude of the pooled effect estimates. Consequently, although the overall associations were consistent, the results should not be interpreted as supporting a universal threshold for clinical decision-making. Future prospective studies should evaluate these biomarkers within multivariable prognostic models and establish clinically relevant, population-specific cut-off values before their routine implementation in clinical practice.

This study provided a comprehensive synthesis of the available evidence on the prognostic value of NLR and PLR in patients with DFU, incorporating data from a relatively large number of studies across diverse populations. The inclusion of multiple clinically relevant outcomes offers a broad assessment of the utility of these biomarkers in different aspects of disease progression. In addition, the use of quantitative meta-analytic techniques, including pooled effect sizes and diagnostic accuracy measures, improves the reliability of the results and allows for a more accurate estimation of the relationships.

However, several limitations should be acknowledged. Some analyses showed high heterogeneity, which may reflect differences in baseline patient characteristics and comorbidities. Furthermore, the relatively small number of studies available for certain outcomes may reduce the reliability of pooled estimates and limit the generalizability of the findings. Another important limitation is the variability in the prognostic thresholds reported for both NLR and PLR across the included studies. Threshold variability may have contributed to the between-study heterogeneity observed in several analyses. Future prospective studies should evaluate these biomarkers to determine clinically relevant cut-off values, preferably within multivariable prognostic models that incorporate established clinical and wound-related risk factors.

## Conclusions

This systematic review and meta-analysis demonstrated that inflammatory hematological ratios (NLR and PLR) are significantly associated with adverse outcomes in patients with DFUs. Both NLR and PLR showed meaningful relationships with amputation, osteomyelitis, and mortality, with the evidence for NLR appearing more consistent across the included studies. These findings suggest that NLR and PLR may serve as useful risk-associated biomarkers to complement clinical assessment. However, the observed associations should not be interpreted as definitive evidence of prognostic performance, as comprehensive prognostic evaluation requires assessment of discrimination, calibration, and external validation. These results affirm the potential of these markers as feasible and affordable tools for the early identification of risks in medical practice. Further well-designed prospective studies are needed to confirm these associations and to evaluate the incremental value of NLR and PLR as components of multivariable risk assessment models that incorporate established clinical and wound-related prognostic factors.

## Appendices

Appendix 1

**Table 2:** Search strategy

Database	Search terms
PubMed	(("platelet to lymphocyte ratio"[Title/Abstract] OR "platelet-lymphocyte ratio"[Title/Abstract] OR PLR[Title/Abstract] OR "neutrophil to lymphocyte ratio"[Title/Abstract] OR "neutrophil-lymphocyte ratio"[Title/Abstract] OR NLR[Title/Abstract])) AND (("diabetic foot ulcer"[Title/Abstract] OR DFU[Title/Abstract] OR "diabetic foot"[Title/Abstract] OR "diabetic septic foot"[Title/Abstract] OR "foot ulcer"[Title/Abstract]))
Web of Science	TS=((("platelet to lymphocyte ratio" OR "platelet-lymphocyte ratio" OR PLR OR "neutrophil to lymphocyte ratio" OR "neutrophil-lymphocyte ratio" OR NLR)) AND TS=( (("diabetic foot ulcer" OR DFU OR "diabetic foot" OR "diabetic septic foot" OR "foot ulcer")))
Embase	(("platelet to lymphocyte ratio":ti,ab OR "platelet-lymphocyte ratio":ti,ab OR plr:ti,ab OR "neutrophil to lymphocyte ratio":ti,ab OR "neutrophil-lymphocyte ratio":ti,ab OR nlr:ti,ab)) AND (("diabetic foot ulcer":ti,ab OR dfu:ti,ab OR "diabetic foot":ti,ab OR "diabetic septic foot":ti,ab OR "foot ulcer":ti,ab))
ProQuest	(ab,ti("platelet to lymphocyte ratio" OR "platelet-lymphocyte ratio" OR PLR OR "neutrophil to lymphocyte ratio" OR "neutrophil-lymphocyte ratio" OR NLR)) AND (ab,ti("diabetic foot ulcer" OR DFU OR "diabetic foot" OR "diabetic septic foot" OR "foot ulcer"))

**Table 3:** GRADE certainty of evidence for the association between NLR/PLR and DFU outcomes AUC: area under the curve; NLR: neutrophil-to-lymphocyte ratio; PLR: platelet-to-lymphocyte ratio; SMD: standardized mean difference; DFU: diabetic foot ulcer

Outcome	Marker	Effect estimate (95% CI)	Risk of bias	Inconsistency	Imprecision	Publication bias	Upgrading factor	Certainty of evidence
Amputation	NLR	SMD 0.78 (0.29-1.28)	Not serious	Serious	Not serious	Not assessed	Large effect	Low
Amputation	PLR	SMD 0.29 (0.09-0.49)	Not serious	Not serious	Serious	Not assessed	None	Low
Osteomyelitis	NLR	SMD 0.31 (0.13-0.48)	Not serious	Not serious	Not serious	Not assessed	None	Low
Osteomyelitis	PLR	SMD 0.40 (0.22-0.57)	Not serious	Not serious	Not serious	Not assessed	None	Low
Mortality	NLR (AUC)	0.83 (0.67-0.99)	Not serious	Not serious	Not serious	Not assessed	Large effect	Moderate
Mortality	PLR (AUC)	0.75 (0.54–0.95)	Not serious	Not serious	Not serious	Not assessed	Large effect	Moderate

**Table 4:** Summary of leave-one-out sensitivity analysis results AUC: area under the curve; NLR: neutrophil-to-lymphocyte ratio; PLR: platelet-to-lymphocyte ratio; SMD: standardized mean difference

Outcome	Biomarker	Pooled estimate	Range of estimates	I² range	Interpretation
Amputation	NLR (SMD)	0.29 (95% CI 0.09-0.49)	0.22-0.39	0-58.2%	Significant in all iterations
Osteomyelitis	NLR (SMD)	0.31 (95% CI 0.13-0.48)	0.25-0.37	0%	Significant in all iterations
Amputation	NLR (SMD)	0.78 (95% CI 0.29-1.28)	0.55-0.87	91.4-93.5%	Significant in all iterations
Mortality	PLR (AUC)	0.75 (95% CI 0.54-0.95)	0.64-0.82	63.1-98.9%	Significant in all iterations
Mortality	NLR (AUC)	0.83 (95% CI 0.67-0.99)	0.76-0.91	88.9-98.4%	Significant in all iterations
Osteomyelitis	PLR (SMD)	0.40 (95% CI 0.22-0.57)	0.34-0.45	0%	Significant in all iterations
